# Updating the fatty acid profiles of retail bovine milk in China based on an improved GC-MS method: implications for nutrition

**DOI:** 10.3389/fnut.2023.1204005

**Published:** 2023-05-25

**Authors:** Meiqing Chen, Fengen Wang, Xufang Wu, Boxue Si, Junyu Pan, Nan Zheng, Yangdong Zhang, Jiaqi Wang

**Affiliations:** ^1^Key Laboratory of Quality and Safety Control for Milk and Dairy Products of Ministry of Agriculture and Rural Affairs, Institute of Animal Sciences, Chinese Academy of Agricultural Sciences, Beijing, China; ^2^Milk and Milk Products Inspection Center of Ministry of Agriculture and Rural Affairs, Institute of Animal Sciences, Chinese Academy of Agricultural Sciences, Beijing, China; ^3^Institute of Quality Standard and Testing Technology for Agro-Products, Shandong Academy of Agricultural Sciences, Jinan, China

**Keywords:** gas chromatography-mass spectrometry, fatty acid profile, retail milk survey, dietary intakes, human health

## Abstract

The importance of food components to potential benefits and risks to human health is gradually being consumer awareness. Milk is an important part of the lipid content of the human diet, and there are few detailed reports on the fatty acid (FA) profiles of retail milk. In the study, we developed a gas chromatography–mass spectrometry (GC-MS) method to simultaneously determine 82 FAs, including 11 even-chain saturated FAs, 10 odd-chain saturated FAs, 9 branched-chain saturated FAs, 30 monounsaturated FAs, and 22 polyunsaturated FAs; this was applied to analyze samples (186 samples) of commercially available milk from 22 provinces throughout China and to evaluate the nutritional value of these samples based on FA-related indices. The results showed that the overall composition of milk FAs among the different regions was numerically similar, and minor FAs showed few differences. When considering the retail milk FA composition and dairy fat intake in China, regional variations have a limited impact on FA consumption. Moreover, milk accounts for approximately one-third and <10% of the maximum recommended intake of saturated FAs and trans-FAs in consumer diets, respectively. This study provides an updated report on the composition of FAs and the nutritional value of retail milk across China, which can serve as a reference for producers for future research on regulating milk FAs, for consumers to select milk, and for nutrition departments to formulate relevant nutritional guidance recommendations.

## 1. Introduction

Milk, which is rich in several essential nutrients such as fat, protein, and minerals, is an important source of bioactive natural ingredients (1). Milk has gradually become an indispensable food in the daily diet of humans, with annual per capita consumption of dairy products increasing by 36.3% in the past decade in China, from 31.1 to 42.3 kg (2). Fatty acids (FAs) are important components of milk fat and are linked to various potential benefits and risks to human health. Previous studies have shown that unsaturated fatty acids (UFAs) reduce hypercholesterolemia and the risk of cardiovascular diseases (CVD), whereas saturated fatty acids (SFAs) and trans-FAs (TFAs) have opposite effects (3–5). Milk typically contains a high proportion of SFAs. However, not all SFAs are equal, and they can negatively affect human health. Recent scientific studies have reported that odd- and branched-chain fatty acids (OBCFA) have positive effects on CVD, cancer, obesity, and inflammation, although they are a category of SFA (6). Ruminant TFA-like vaccenic acid was not associated with an increased risk of CVD. Milk is the only source of unique rumen microorganism-synthesized FAs such as OBCFA and vaccenic acid (VA, C18:1 t11). Moreover, milk contains UFAs, such as conjugated linoleic acid (LA), omega-3 (n-3) UFA, omega-6 (n-6) UFA, and omega-7 (n-7) UFA, which have been shown to provide potential benefits, including CVD prevention and anti-cancer, anti-inflammatory, and anti-oxidative effects (7–9). Since FAs with structural differences present distinct effects on biological functions, it may be more important to pay attention to the health benefits of individual FAs compared to FA groups. Therefore, it is imperative to comprehensively characterize the FA composition of milk to evaluate its nutritional health effects.

Over 400 FAs are thought to be present in bovine milk (10); however, most FAs, especially those in low abundance, have not been quantified in the previous report (11). For example, in the face of the prevalent co-elution of complex geometric and positional isomers of UFA in milk, we were unable to determine whether C18:1 UFA isomer or C18:2 UFA isomer co-eluted with other FAs in milk (12). Owing to the high complexity of FAs in species and their abundance, the simultaneous determination of multiple components is a challenge (13). For FA determination, it is a common practice to convert FAs to methyl ester (FAME) using methanol, which is then analyzed using a gas chromatograph (GC) equipped with a flame ionization detector (FID) or mass spectrometry (MS). With an FID, the identification and quantification of low-abundance and co-eluted FAs from chromatogram peaks are challenging. In contrast, MS has better selectivity and sensitivity for monitoring specific ions, as well as good accuracy for FA determination. Several reports have discussed the significant improvements in the development of a high-throughput method for the determination of milk FAs by MS (14–16); ~70 FAs except the co-eluted octadecenoic acid were accurately quantified by Wang et al. (17). Focusing on the challenge of effective identification of FAs, appropriate chromatographic procedures must be selected to achieve effective chromatographic separation of as many analytes as possible according to their properties (18). For the accurate quantification of FAs, appropriate derivatization treatments must be selected to improve the response of the analytes. Hence, it is necessary to improve the resolution and sensitivity of detection to establish a high-throughput FA analysis method, which is conducive to the comprehensive characterization of milk FA composition.

Our current study showed that it is challenging for consumers to comprehend the relevance of milk FA composition to human health because they know little about how milk is produced and do not understand the significance of milk FA profile. For FA profiles to be easier to understand, researchers have proposed FA indices such as the atherogenic index (AI) and thrombogenic index (TI), which assess the effects of food on cardiovascular health (CVH) and quantify the relevance of FA composition to human health (19, 20). Moreover, the assessment of dietary intake, including FA intake, is an important tool for monitoring the nutritional status of a population. Current nutritional recommendations suggest limiting the SFA and TFA consumption to <10 and 1% of energy intake, respectively, and increasing the dietary UFA consumption as much as possible (21). The composition of milk in retail markets across the United States (18), United Kingdom (22), and Korea (23) has been systematically researched, whereas few studies have been conducted in China. Previous studies have shown that milk FA profiles may vary greatly among countries and regions. Therefore, it is necessary to set the appropriate dietary intake of people in one's own country or region according to the assessment of milk FA composition.

Limited information is available on the relevance of FA profile to nutritional value in retail milk surveys across China, which may affect consumer decisions and human health. Therefore, the aims of this study were to (i) comprehensively characterize the FA composition of retail milk across China using an improved high-throughput FA determination method and (ii) evaluate the potential nutritional implications of FA indices of retail milk and FA intake.

## 2. Materials and methods

### 2.1. Milk samples

Milk samples (*n* = 186) were collected from supermarkets in four regions (Northeast China and Inner Mongolia, North China, Northwest China, and South China) throughout China in 2021, which included 44 brands and 22 provinces (Supplementary Table S1). The samples were whole and pasteurized bovine milk and were stored at −20°C after collection until analysis. In addition, skim milk powder from local markets was used in the recovery experiments for method validation.

### 2.2. Chemicals and reagents

High-performance liquid chromatography-grade n-hexane, methanol, and isopropanol were obtained from Fisher Scientific (Fair Lawn, NJ, USA). NaOH (purity ≥95%), anhydrous sodium sulfate (purity ≥99%), and acetyl chloride (purity >99.5%) were from Macklin (Shanghai, China). Ultrapure water was obtained using a Milli-Q purification system (Millipore, Bedford, MA, USA).

The following standards were used: FA solution GLC 617 (Anpel, Shanghai, China) and FAME solution GLC 674 (Nu-Chek Prep, Inc., Elysian, MN, USA); 6 iso-FAME and 3 anteiso-FAME individual standards were obtained from Larodan (Malmo, Sweden). The C22:3 c13c16c19 FAME individual standard and C17:0 ethyl ester (C17:0 EE) used as internal standards (IS) were obtained from AccuStandard (New Haven, Connecticut, USA). The individual standards of C18:1 c12, C18:4 c6c9c12c15, C19:1 c10, and C20:1 c8 FAMEs, as well as C18:2 c9c11 and C18:2 t9t11 FA individual standards, were obtained from Cayman (Ann Arbor, Michigan, USA). The C5:0, C7:0, C9:0, and C19:0 FAME individual standards were obtained from Dr. Ehrenstorfer (Augsburg, Germany). The C10:1 c3, C10:1 c4, C10:1 c9, C12:1 c5, C12:1 c11, and C16:1 c7 FA individual standards were obtained from Macklin (Shanghai, China). Linoleic acid methyl ester mix Certified Reference Material 47791 and C18:2 c9t11 and C18:2 t10t12 FA individual standards were purchased from Sigma-Aldrich (St. Louis, MO, USA). The triacylglycerol (TAG) individual standard of C11:0 was obtained from Nu-Check Prep (Elysian, MN, USA).

### 2.3. Sample pretreatment

Based on our previous study, a sample pretreatment procedure was developed, including the extraction of lipids from milk and the conversion of FA into FAME (17, 24). Frozen milk samples were first pre-warmed at 40°C in a water bath and then shaken carefully to homogenize them. To extract milk lipids, 2 ml milk samples were mixed with 4 ml of a solution of n-hexane/isopropanol (v/v, 3/2), vortexed, and centrifuged. The upper n-hexane was collected, followed by another extraction with n-hexane, and all of the extracted upper n-hexane were collected and mixed together. Combined n-hexane was mixed with 2 ml solution of methanolic NaOH (2%) with heating at 50°C for 20 min, followed by 2 ml solution of acetyl chlorocarbinol (10%) with heating at 90°C for 150 min for methylation. After cooling to room temperature, 5 ml of ultrapure water was added to the mixture. The upper n-hexane phase was then extracted and diluted. Anhydrous sodium sulfate (0.5 g) was then added, and the mixture was vortexed for 30 s for further dewatering. The supernatant was mixed with an internal standard, diluted with n-hexane, and subsequently analyzed using gas chromatography–mass spectrometry (GC-MS).

### 2.4. GC-MS analysis

Analyses of FAMEs were performed using an Agilent 7890A GC equipped with a 7000 B MS detector system (Agilent Technologies, Santa Clara, California, USA). FAMEs were separated using an Agilent capillary column CP-Sil 88 (100 m × 0.25 mm × 0.20 μm). The proposed method was improved using an applicable GC oven program (Table 1) and by performing in the selected ion monitoring (SIM) mode with 19-time windows (Table 2). Four characteristic ions were selected for the qualification and quantification of each analyte and IS, and the lowest signal-to-noise ratio (S/N) was designated as the quantitative ion. Qualitative analysis of individual FAME in milk samples was performed by comparing their retention times and characteristic ions with those of the corresponding FAME standards. Quantitative analysis of individual FAME was carried out by external standard calculation and IS calibration, that is, using IS calibration, the value of the curve regression equation for each FAME standard was calculated. The FA values were calculated using the stoichiometric factors for the conversion of FAME into FA. The FA composition results are expressed as g/100 g FA.

**Table 1:** GC-MS parameters of the proposed method for determination of 82 fatty acid methyl ester.

**Items**	**Set parameters**
GC-MS apparatus	Agilent 7890A GC−7000B MS
Column	CP-Sil 88 (100 m × 0.25 mm × 0.20 μm)
Injection volume	1 μl
Split ratio	20:1
Carrier gas	Helium
Carrier gas pressure	38 psi
Inlet temperature	250°C
Oven temperature	120°C (10 min) → 3°C/min → 180°C (30 min) → 15°C/min → 210°C (13 min) → 10°C/min → 230°C (13 min)
Transfer line temperature	250°C
MS ion source temperature	230°C
MS quadrupole temperature	150°C
Solvent delay	9 min
Ionization energy	70 eV

**Table 2:** MS SIM parameters of the proposed method for determination of 82 fatty acid methyl ester.

**No**.	**FAME**	**Group**	**RT (min)**	**Quantification ion (*m*/*z*)**	**Qualification ion (** ***m*** **/** ***z*** **)**			**Dwell time (ms)**
1	C4:0	1	9.188	74	71	87	59	11
2	C5:0		9.695	74	85	57	87	
3	C6:0		10.436	74	87	99	101	
4	C7:0		11.503	74	87	113	101	
5	C8:0	2	13.021	74	87	127	115	12
6	C9:0		15.054	74	87	141	129	
7	C10:0		17.643	74	87	143	155	
8	C10:1 c4	3	18.584	74	110	152	96	12
9	C10:1 c3		19.585	74	110	152	96	
10	C10:1 c9		20.340	74	110	152	96	
11	C11:0		20.630	74	87	143	169	
12	C12:0		23.794	74	87	143	171	
13	C12:1 c5	4	25.245	180	96	138	74	14
14	C13:0 iso		25.407	185	87	143	74	
15	C12:1 c11		25.588	180	96	138	74	
16	C13:0 anteiso		25.966	185	87	143	74	
17	C13:0		26.961	185	87	143	74	
18	C14:0 iso	5	28.539	74	87	143	199	12
19	C14:0		30.031	74	87	143	199	
20	C15:0 iso		31.564	256	87	74	143	
21	C14:1 t9		31.657	208	74	166	124	
22	C15:0 anteiso		32.141	256	87	143	74	
23	C14:1 c9		32.425	208	166	74	124	
24	C15:0		33.099	256	87	143	74	
25	C16:0 iso	6	34.731	270	87	143	74	12
26	C15:1 t10		34.861	222	96	138	180	
27	C15:1 c10		35.681	222	96	138	180	
28	C16:0		36.392	270	87	143	74	
29	C16:1 t9	7	37.988	194	152	236	110	12
30	C17:0 iso		38.153	284	87	143	74	
31	C16:1 c7		38.284	194	152	236	110	
32	C16:1 c9		38.723	194	152	236	110	
33	C17:0 anteiso		38.853	284	87	143	74	
34	C17:0		39.980	74	87	143	284	
IS	C17:0 FAEE	8	41.446	88	101	157	298	10
35	C17:1 t10		41.836	250	97	208	166	
36	C18:0 iso		42.018	298	87	143	74	
37	C17:1 c10		42.675	250	97	208	166	
38	C18:0		44.164	298	87	143	74	
39	C18:1 t6	9	45.931	264	97	222	180	12
40	C18:1 t9		46.101	264	97	222	180	
41	C18:1 t11		46.354	264	97	222	180	
42	C18:1 c6		46.714	264	97	222	180	
43	C18:1 c9		46.929	264	97	222	180	
44	C18:1 c11		47.384	264	97	222	180	
45	C18:1 c12		47.748	264	97	222	180	
46	C19:0		49.222	312	87	74	143	
47	C18:2 t9t12	10	49.762	294	81	95	263	12
48	C18:2 c9t12		50.866	294	81	95	263	
49	C18:2 t9c12		51.328	294	81	95	263	
50	C19:1 t7		51.371	278	97	111	236	
51	C19:1 t10		51.586	278	97	111	236	
52	C18:2 c9c12		51.993	294	81	95	263	
53	C19:1 c10		52.405	278	97	111	236	
54	C20:0	11	55.558	74	326	87	143	9
55	C18:3 c6c9c12		56.358	79	93	121	292	
56	C20:1 t11		58.392	250	97	208	292	
57	C18:3 c9c12c15		59.287	79	93	121	292	
58	C20:1 c8		59.016	250	97	208	292	
59	C20:1 c11		59.642	250	97	208	292	
60	C18:2 c9t11	12	60.776	294	81	95	263	12
61	C18:2 t10c12		61.567	294	81	95	263	
62	C18:2 c9c11		61.935	294	81	95	263	
63	C21:0		62.218	340	74	87	143	
64	C18:4 c6c9c12c15	13	62.935	221	91	79	161	12
65	C18:2 t9t11		62.948	294	81	95	263	
66	C20:2 c11c14		63.993	81	95	322	291	
67	C22:0	14	66.482	354	74	87	143	12
68	C20:3 c8c11c14		66.792	79	95	108	320	
69	C22:1 t13	15	68.165	236	97	111	320	10
70	C20:3 c11c14c17		68.598	79	95	108	320	
71	C22:1 c13		68.884	236	97	111	320	
72	C20:4 c5c8c11c14		69.041	79	91	105	119	
73	C23:0	16	71.415	87	74	368	143	12
74	C22:2 c13c16		73.468	95	319	109	350	
75	C20:5 c5c8c11c14c17	17	74.862	79	91	105	119	12
76	C24:0		76.241	87	74	382	143	
77	C22:3 c13c16c19	18	77.963	79	108	121	261	12
78	C24:1 c15		78.164	264	97	111	348	
79	C22:4 c7c10c13c16	19	78.771	79	91	105	150	20
80	C22:5 c4c7c10c13c16		80.407	79	91	105	119	
81	C22:5 c7c10c13c16c19		82.944	79	91	105	119	
82	C22:6 c4c7c10c13c16c19		84.852	79	91	105	119	

FAME, fatty acid methyl ester; RT, retention time; IS, internal standard.

### 2.5. Method validation

Sensitivity, linearity, accuracy, and precision were involved in validating the method (25). Sensitivity was calculated from the concentration with an S/N ratio of 10 and expressed as the limit of quantitation (LOQ) (15). Linearity was assessed using the coefficient of determination (*R*^2^) of the standard curves of each FAME at five different concentration levels (concentration ratio between FAME and IS vs. the peak area ratio). Recovery experiments were spiked with three different concentrations of FA solution (GLC 617) and triacylglycerol (C11:0) in a solution of skim milk powder, and the results were used to demonstrate the accuracy. The intra- and inter-day precisions were determined by testing six parallel samples three times within a day and once a day for three consecutive days, respectively.

### 2.6. Statistical analysis

The peak areas were obtained using the Agilent MassHunter Workstation (Agilent Technologies, Santa Clara, California, USA). The results were preliminarily sorted using Microsoft Excel 2019. Before significance analysis, the Kolmogorov–Smirnov test was applied to determine whether the results coincided with the normal distribution obtained using the univariate procedure in SAS 9.4. The Kruskal–Wallis test was performed on the FA data that did not comply with the normal distribution using the NPAR1WAY procedure in SAS 9.4. Multiple comparisons were performed on the transformed RANK of the original data with Duncan's method using the generalized linear model (GLM) procedure in SAS 9.4. A *P*-value of < 0.05 was considered statistically significant.

Some FA indices, such as n-6/n-3 polyunsaturated fatty acid (PUFA), PUFA/SFA ratio (P/S), AI, TI, health-promoting index (HPI), and hypo/hypercholesterolemic ratio (h/H), were calculated as described previously (20), as follows:


$$\begin{array}{l} \text{n}-6/\text{n}-3\text{ PUFA}=\Sigma \text{n}-6\text{ PUFA}/\Sigma \text{n}-3\text{ PUFA};\\ \text{ P}/\text{S}=\text{ΣPUFA}/\text{ΣSFA};\\ \text{ AI}=(\text{C}12:0+4\times \text{C}14:0+\text{C}16:0)/\\ \;(\text{ΣMUFA}+\text{ΣPUFA});\\ \text{ TI}=(\text{C}14:0+\text{C}16:0+\text{C}18:0)/\\ \;[(0.5\times \text{ΣMUFA}+0.5\times \text{ΣPUFA }(\text{n}6)+\\ \;3\times \text{ΣPUFA }(\text{n}3))+(\text{n}3)/(\text{n}6)];\\ \text{ HPI}=(\text{ΣMUFA}+\text{ΣPUFA})/(\text{C}12:0\\ \;+4\times \text{C}14:0+\text{C}16:0);\\ \text{ h}/\text{H}=(\text{C}18:1\text{n}9+\text{C}18:2\text{n}6+\text{C}20:4\text{n}6\\ \;+\text{C}18:3\text{n}3+\text{C}20:5\text{n}3+\text{C}22:5\text{n}3\\ \;+\text{C}22:6\text{n}3)/(\text{C}14:0+\text{C}16:0). \end{array}$$

To calculate the FA intake from retail milk, we assumed that the FA composition of all dairy products produced in China was consistent with that of the milk samples analyzed in this study. The FA intake via retail milk consumption was estimated (26, 27) as follows:

FA intake (mg/d) = 300 (milk intake, g/d) × 1, 000 × 3.8 (contribution of fat from whole milk, %) ÷100 × 93.3 (the proportion of FA in milk fat, %) ÷100 × milk FA concentration (the proportion of FA in total FA, %) ÷100.

## 3. Results and discussion

### 3.1. Method optimization

**Figure 1:**
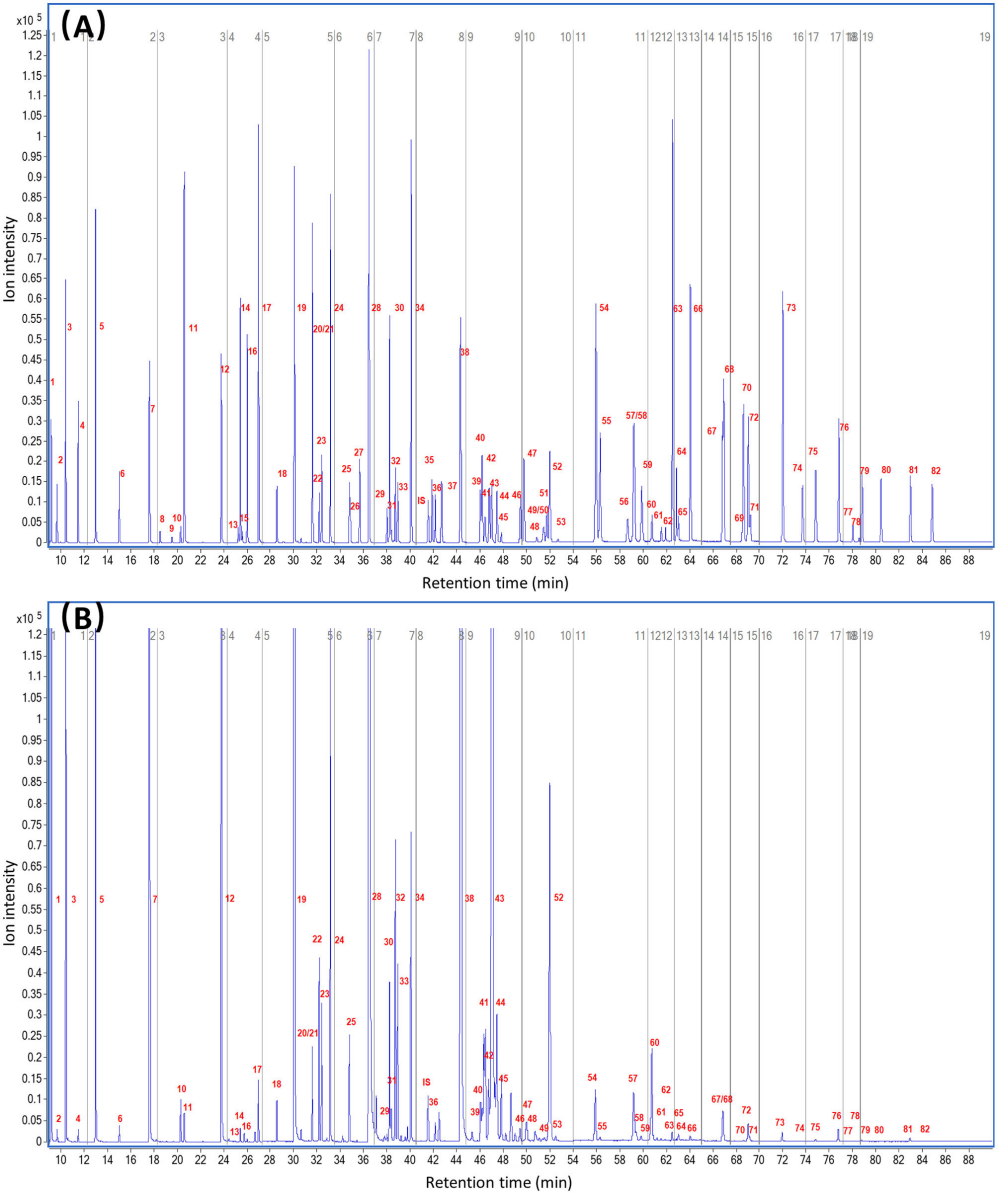
Total ion chromatogram of fatty acid methyl ester by GC-MS. **(A)** 82 fatty acid methyl ester standards. **(B)** Fatty acid methyl ester from raw cow milk. The number of fatty acid methyl ester in Table 2 is labeled on the peaks.

Using the proposed method, 82 FAs were detected simultaneously by chromatographic and spectrographic optimization, including 11 ECSFAs, 10 OCSFAs, nine BCSFAs, 30 MUFAs, and 22 PUFAs (Figure 1).

**Figure 2:**
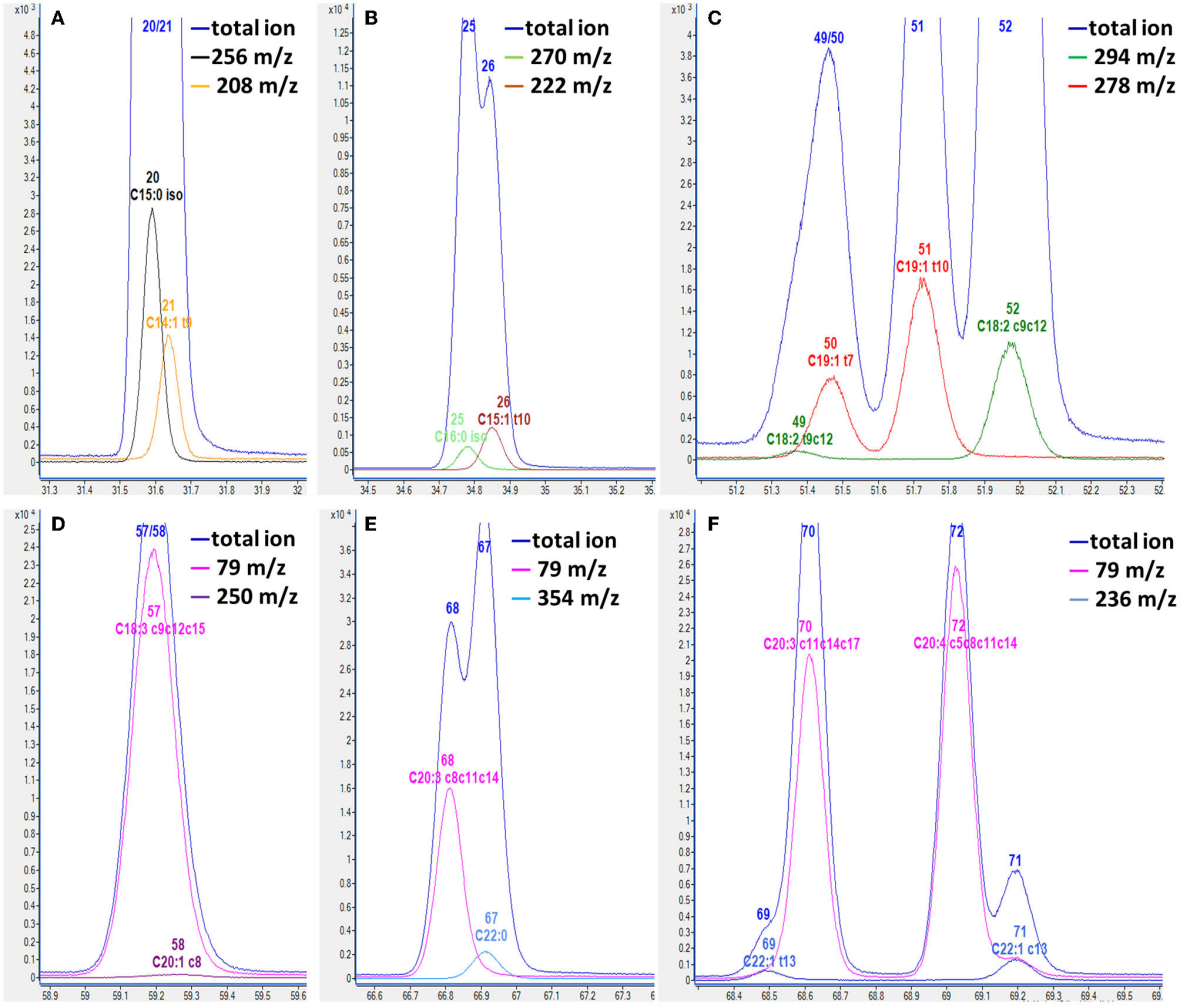
Overlapped chromatogram of total selected ion and individual selected ion of fatty acid methyl ester by GC-MS. **(A)** C15:0 iso and C14:1 t9; **(B)** C16:0 iso and C15:1 t10; **(C)** C18:2 t9c12 and C19:1 t7; **(D)** C18:3 c9c12c15 and C20:1 c8; **(E)** C22:0 and C20:3 c8c11c14; **(F)** C22:1 t13 and C20:3 c11c14c17, C22:1 c13 and C20:4 c5c8c11c14. The number of fatty acid methyl ester in Table 2 is labeled on the peaks.

Regarding chromatographic separation, the capillary column and oven temperature program were optimized by virtue of the differences in boiling point and polarity among the abovementioned FAs. Several capillary columns, including DB-WAX and CP-Sil 88, were attempted, and most of the FAME peaks presented less co-elution, excellent peak symmetry, and rational retention times on the CP-Sil 88 column; therefore, we selected it for further study. It was noticed that an oven temperature of ~180°C gave better resolution among FAMEs with <C18, and an oven temperature of ~210°C gave better resolution among FAMEs with >C18. Separation behaviors of cis- and trans-isomers of C18:1 are greatly affected by column and oven temperatures (28, 29). Thus, the column temperature was divided into three temperature change stages by setting an appropriate rate of increase in the oven temperature to achieve baseline separation of most FAMEs in the total ion chromatogram (TIC; Figure 1). Three types of co-elution problems were observed in the TIC which made the quantification of co-eluted FA almost impossible without the SIM mode. (i) the co-elution of methyl BCFA and MUFA, as shown in Figures 2A, B; (ii) the co-elution of methyl MUFA and PUFA, as shown in Figures 2C–F; (iii) the co-elution of methyl PUFA and SFA, as shown in Figure 2E. In previous reports, the problem of co-elution is common in the analysis of fatty acids by gas chromatography, including the interference of methyl BCFA and MUFA and the co-elutions of cis- and trans-isomers of both C16:1 and C18:1 (12, 30). To eliminate interference between the peaks of partial and complete co-elution, we analyzed the characteristics of the unique fragment ions of these co-elutions using SIM (14). Identification and quantification were based on the SIM mode shown in Table 2. For example, C15:0 iso methyl and C14:1 t9 methyl co-eluted in the TIC and were distinguished by specific fragment ions at 256 and 208 *m*/*z*, respectively, in the extracted ion chromatogram, which provided good resolution (Figure 2A). Similarly, the SIM parameters for the analysis of other co-eluted FAMEs in the TIC were also optimized (Figures 2A–F).

The results of the method validation are shown in Supplementary Tables S2, S3. The limit of quantitation (LOQ) of FAMEs ranged from 1.9 to 990.1 μg/L corresponding to 0.0439 μg/ml to 23.6832 μg/ml of FAs from milk samples. The regression equations of all the FAME standard curves covered a sufficiently wide linear quantitative range, with determination coefficients >0.9991. Recoveries of FAs ranged from 81.4 to 108.3%, while those of TAGs were 101.9 to 106.3%. Intra-day and inter-day relative standard deviations (RSDs) were <5.0% based on the determined FAs in the milk samples.

In general, method optimization involves the choice of polar column, column temperature program, and characteristic ions that influence separation efficiency along with the accurate determination of all FAs from bovine milk samples, particularly UFAs that have received little or no attention in the FA analyses, including n-1, n-3, n-5, n-6, n-7, n-9, and n-12 UFAs. Method validation showed that the proposed method presented competitive sensitivity, suitable linearity, acceptable accuracy, and precision for the determination of milk FAs. Thus, a high-throughput GC-MS method was applied to determine milk FAs, with good separation in the chromatogram and good resolution in the SIM mode, which is the basis of a comprehensive understanding of milk FA composition.

### 3.2. Milk FA profiles

**Figure 3:**
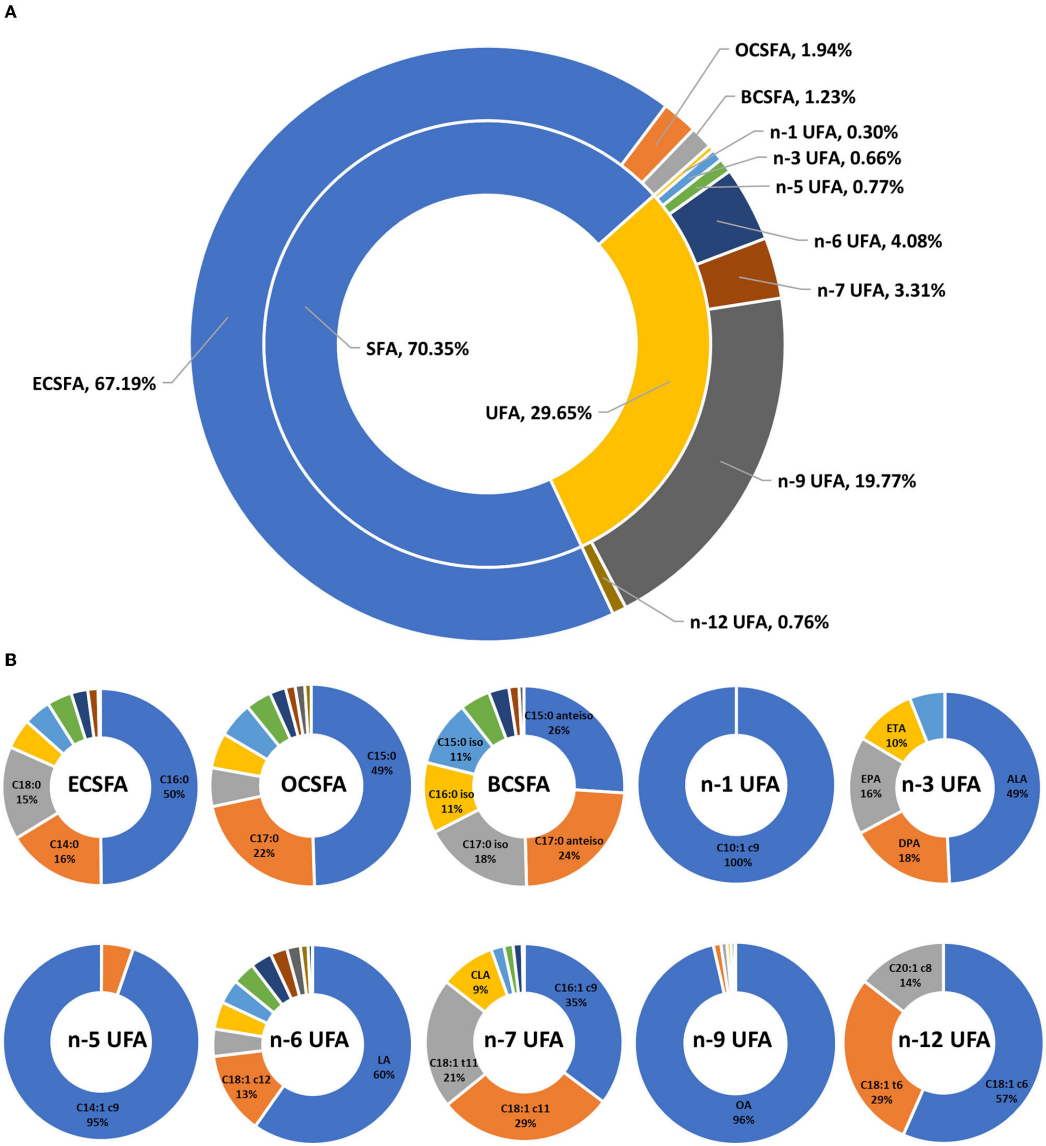
Fatty acid profiles of bovine retail milk in China. **(A)** Contribution (%) of each fatty acid group to total fatty acid of milk. **(B)** Contribution (%) of individual fatty acid to total corresponding fatty acid group of milk. SFA, saturated fatty acid, C4:0–C24:0 (including iso and anteiso). ECSFA, even-chain saturated fatty acid, C4:0–C24:0. OCSFA, odd-chain saturated fatty acid, C5:0–C23:0. BCSFA, branched-chain saturated fatty acid, C13:0–C18:0 iso and anteiso. UFA, unsaturated fatty acid, C10:1–C22:6. n-1 UFA, omega-1 unsaturated fatty acid, contains one or more than one carbon-to-carbon double bond with the first double bond placed on the first carbon atoms counting from the methyl end. n-3 UFA, omega-3 unsaturated fatty acid. n-5 UFA, omega-5 unsaturated fatty acid. n-6 UFA, omega-6 unsaturated fatty acid. n-7 UFA, omega-7 unsaturated fatty acid. n-9 UFA, omega-9 unsaturated fatty acid. n-12 UFA, omega-12 unsaturated fatty acid. OA, C18:1 c9. LA, linoleic acid, C18:2 c9c12. CLA, conjugated linoleic acid, C18:2 c9t11. ALA, α-linolenic acid, C18:3 c9c12c15. ETA, eicosatrienoic acid, C20:3 c11c14c17. EPA, eicosapentaenoic acid, C20:5 c5c8c11c14c17. DPA, docosapentaenoic acid, C22:5 c7c10c13c16c19. DHA, docosahexaenoic acid, C22:6 c4c7c10c13c16c19.

The FA profiles of bovine retail milk from China are shown in Figure 3. The ratio of SFA to UFA in retail milk in China is ~7:3 (Figure 3A). Previous studies have reported that the ratio of SFA and UFA in bovine retail milk in the United Kingdom ranged from 73:27 to 68:32 (22, 31), while the ratio in the United States ranged from 70:30 to 68:32 (18). This is inconsistent with our findings because FA composition is affected by many factors, including diet and season (32, 33). In addition, some studies did not consider as many FAs as possible and only included the high content ones in the analysis; therefore, the results of these studies were biased compared to our results. In addition to the major ECSFAs, OCSFA, and BCSFA are important components of milk SFAs. C14:0, C16:0, and C18:0 were the most abundant ECSFAs, whereas C15 and C17 FAs with straight and branched chains were the most abundant OCSFAs (Figure 3B). This observation is consistent with that of other studies and is mainly determined by the synthesis of milk FAs in dairy cows, primarily from the *de novo* synthesis of FAs with carbon chain length <C16 and the intake of FAs with carbon chain length >C16 from the blood in the mammary glands (34, 35). n-9, n-6, and n-7 UFAs were the main components of milk UFAs, whereas other UFAs were relatively minor, accounting for <1% of the total FAs (Figure 3A). In our study, oleic acid (OA, C18:1 c9) was the most dominant n-9 UFA in milk, accounting for 96% of the total n-9 UFAs. LA and C18:2 c12 were the main n-6 UFAs in milk, accounting for ~60 and 13% of the total n-6 UFAs, respectively. Alpha-linolenic acid (ALA, C18:3 c9c12c15), docosapentaenoic acid n-3 (DPA n-3, C22:5 c7c10c13c16c19), eicosapentaenoic acid (EPA, C20:5 c5c8c11c14c17), and eicosatrienoic acid (ETA, C20:3 c11c14c17) were the main n-3 UFAs in milk, accounting for 49%, 18%, 16%, and 10% of the total n-3 UFA, respectively. This is because LA (C18:2 c9c12) and ALA are the precursors of n-6 UFA and n-3 UFA synthesis, respectively, in dairy cows, and these precursors synthesize the corresponding UFAs through carbon chain elongation and desaturation (36). In addition, we found that C10:1 c9 was the only n-1 UFA in milk, and C14:1 c9, C16:1 c9, and C18:1 c6 were the main n-5, n-7, and n-12 UFAs in milk, respectively. Previous studies have reported the functions of n-9, n-6, and n-3 UFAs; therefore, the content of these UFAs in milk has been widely studied. The results of our study provide information on the content of other minor UFAs in milk, which could provide reference data for subsequent research on the function of milk FAs.

The FA composition of retail milk across four regions of China is presented in Table 3. Statistical differences were observed in the concentrations of some individual FAs for different geographic regions, including C4:0, C15:0, C15:0 anteiso, VA, LA, conjugated linoleic acid n-7 (CLA n-7, C18:2 c9t11), ALA, arachidonic acid (ARA, C20:4 c5c8c11c14), and FAs (including OCSFA, BCSFA, n-6 PUFA, n-1 UFA, n-5 UFA, n-6 UFA, n-7 UFA, and n-12 UFA). There were no significant differences in the concentrations of individual FAs, including C16:0, C18:1 c9, n-3 UFA, EPA, DPA, DPA n-6, and FA, including SFA, MUFA, and PUFA. Differences in rumen fermentation due to the diet and management of dairy cows may partly explain the inconsistent content of the abovementioned OBCFA and UFA among the regions (37, 38). In this study, where milk was collected at the retail level, it was not possible to collect detailed information at the farm level, and, therefore, we cannot provide a more detailed explanation of the subtle variations in some FAs. In general, although the minor FAs showed numerically smaller significant differences among the regions, the major FAs mostly showed no significant differences. Thus, the overall composition of milk FAs among different regions was numerically similar, and the minor FAs showed little difference.

**Table 3:** Regional variation in the fatty acid composition of retail milk samples.

**FA (g/100g FA)**	**NEC (*n* = 8)**	**NC (*n* = 51)**	**NWC (*n* = 13)**	**SC (*n* = 114)**	**Mean**	**SEM**	***P*-value^1^**
**Individual FA**							
**SFA** ^2^							
***ECSFA** ^3^*							
C4:0	2.52^c^	3.42^a^	2.90^b^	3.23^a^	3.02	0.037	^**^
C6:0	1.63^b^	2.04^a^	1.73^b^	1.96^a^	1.84	0.021	^**^
C8:0	1.06^b^	1.22^a^	1.09^b^	1.20^a^	1.14	0.011	^**^
C10:0	2.76	2.74	2.73	2.78	2.75	0.022	ns
C12:0	3.38	3.19	3.38	3.29	3.31	0.025	ns
C14:0	11.41^a^	10.74^b^	11.33^a^	10.65^b^	11.03	0.062	^**^
C16:0	34.25	32.58	33.83	33.38	33.51	0.202	ns
C18:0	10.46	10.57	9.99	10.28	10.32	0.070	ns
C20:0	0.08^b^	0.16^a^	0.15^a^	0.16^a^	0.14	0.005	^**^
C22:0	0.05^b^	0.06^ab^	0.07^a^	0.07^a^	0.06	0.002	^*^
C24:0	0.04^b^	0.06^a^	0.07^a^	0.07^a^	0.06	0.003	ns
***OCSFA** ^4^*							
C5:0	0.02^b^	0.04^a^	0.03^a^	0.04^a^	0.03	0.001	^**^
C7:0	0.02^b^	0.02^a^	0.02^a^	0.03^a^	0.02	0.001	^*^
C9:0	0.03^b^	0.05^a^	0.05^a^	0.06^a^	0.05	0.001	^**^
C11:0	0.06^b^	0.08^a^	0.08^a^	0.09^a^	0.08	0.002	^*^
C13:0	0.10^b^	0.12^a^	0.12^a^	0.12^a^	0.11	0.002	^*^
C15:0	0.88^c^	0.95^b^	1.04^a^	0.97^b^	0.96	0.007	^**^
C17:0	0.35^b^	0.46^a^	0.43^a^	0.47^a^	0.43	0.005	^**^
C19:0	0.03^b^	0.04^a^	0.03^ab^	0.04^ab^	0.03	0.002	ns
C21:0	0.08	0.11	0.12	0.11	0.11	0.005	ns
C23:0	0.08	0.14	0.13	0.13	0.12	0.005	ns
***BCSFA** ^5^*							
C13:0 iso	0.01^b^	0.02^ab^	0.02^ab^	0.03^a^	0.02	0.002	^*^
C13:0 anteiso	0.003^b^	0.02^ab^	0.01^ab^	0.03^a^	0.01	0.002	^*^
C14:0 iso	0.05^b^	0.07^a^	0.07^a^	0.08^a^	0.06	0.002	^**^
C15:0 iso	0.10^b^	0.13^ab^	0.12^ab^	0.15^a^	0.13	0.004	^**^
C15:0 anteiso	0.28^b^	0.32^ab^	0.32^ab^	0.35^a^	0.32	0.007	^*^
C16:0 iso	0.11^b^	0.14^a^	0.14^a^	0.16^a^	0.14	0.003	^**^
C17:0 iso	0.20	0.22	0.22	0.25	0.22	0.006	ns
C17:0 anteiso	0.27	0.29	0.29	0.32	0.29	0.007	ns
C18:0 iso	0.02^b^	0.04^ab^	0.04^ab^	0.05^a^	0.04	0.002	^*^
**UFA** ^6^							
*n*−1*UFA*^7^							
C10:1 c9	0.27	0.31	0.30	0.32	0.30	0.004	^*^
C12:1 c11	NA	NA	NA	NA	NA
*n*−3*UFA*^8^							
C18:3 c9c12c15	0.29^b^	0.32^a^	0.35^a^	0.36^a^	0.33	0.007	^**^
C18:4 c6c9c12c15	NA	NA	NA	NA	NA
C20:3 c11c14c17	0.08^a^	0.03^ab^	0.08^b^	0.08^ab^	0.07	0.006	^*^
C20:5 c5c8c11c14c17	0.07	0.13	0.13	0.13	0.11	0.006	ns
C22:3 c13c16c19	NA	0.06	0.05	0.04	0.04	0.004	ns
C22:5 c7c10c13c16c19	0.07	0.13	0.13	0.13	0.12	0.005	ns
C22:6 c4c7c10c13c16c19	NA	NA	NA	NA	NA
*n*−5*UFA*^9^							
C14:1 t9	0.03	0.04	0.03	0.04	0.04	0.002	ns
C14:1 c9	0.63^c^	0.80^a^	0.73^b^	0.77^ab^	0.73	0.007	^**^
C15:1 t10	NA	NA	NA	NA	NA
C15:1 c10	NA	NA	NA	NA	NA
*n*−6*UFA*^10^							
C10:1 c4	NA	NA	NA	NA	NA
C18:1 c12	0.51	0.61	0.52	0.54	0.54	0.012	ns
C18:2 t9t12	0.07^c^	0.27^a^	0.17^b^	0.23^ab^	0.18	0.010	^**^
C18:2 c9t12	0.05	0.06	0.06	0.05	0.05	0.001	ns
C18:2 t9c12	0.05^a^	0.02^b^	0.04^a^	0.03^ab^	0.03	0.002	^*^
C18:2 c9c12	2.51^a^	2.56^a^	2.11^b^	2.50^a^	2.42	0.027	^**^
C18:2 t10c12	NA	NA	NA	NA	NA
C18:3 c6c9c12	0.13	0.15	0.17	0.16	0.15	0.004	ns
C20:2 c11c14	0.09	0.17	0.16	0.16	0.14	0.006	ns
C20:3 c8c11c14	0.12^b^	0.17^a^	0.17^a^	0.17^a^	0.16	0.004	^**^
C20:4 c5c8c11c14	0.12^b^	0.18^a^	0.21^a^	0.21^a^	0.18	0.008	^*^
C22:2 c13c16	NA	NA	NA	NA	NA
C22:4 c7c10c13c16	0.07	0.12	0.10	0.09	0.09	0.007	ns
C22:5 c4c7c10c13c16	0.07	0.12	0.12	0.12	0.11	0.005	ns
*n*−7*UFA*^11^							
C10:1 c3	NA	NA	NA	NA	NA
C12:1 c5	NA^b^	0.005^ab^	0.01^ab^	0.01^a^	0.01	0.001	^*^
C16:1 t9	0.05^c^	0.08^c^	0.07^bc^	0.07^ab^	0.07	0.002	^**^
C16:1 c9	0.92^c^	1.35^a^	1.11^b^	1.28^a^	1.17	0.019	^**^
C17:1 t10	NA	NA	NA	NA	NA
C17:1 c10	NA	NA	NA	NA	NA
C18:1 t11	0.72^ab^	0.81^a^	0.61^b^	0.70^ab^	0.71	0.015	^**^
C18:1 c11	0.72^b^	1.10^a^	0.93^a^	1.08^a^	0.96	0.022	^**^
C18:2 c9t11	0.24^b^	0.36^a^	0.26^ab^	0.33^a^	0.30	0.011	^*^
C18:2 c9c11	0.05^ab^	0.04^c^	0.06^a^	0.05^bc^	0.05	0.001	^**^
C18:2 t9t11	0.05	0.05	0.06	0.05	0.05	0.001	ns
*n*−9*UFA*^12^							
C16:1 c7	0.17	0.20	0.19	0.20	0.19	0.002	ns
C18:1 t9	0.20^b^	0.27^a^	0.24^ab^	0.26^a^	0.24	0.006	^*^
C18:1 c9	20.50	18.35	19.17	18.21	19.06	0.206	ns
C19:1 t10	NA	NA	NA	NA	NA
C19:1 c10	NA	NA	NA	NA	NA
C20:1 t11	NA	NA	NA	NA	NA
C20:1 c11	0.09	0.11	0.16	0.14	0.12	0.006	ns
C22:1 t13	NA	NA	NA	NA	NA
C22:1 c13	0.07	0.14	0.16	0.15	0.13	0.008	ns
C24:1 c15	NA^b^	0.03^a^	0.04^a^	0.02^a^	0.02	0.002	^*^
*n*−12*UFA*^13^							
C18:1 t6	0.15^c^	0.27^a^	0.21^bc^	0.24^ab^	0.22	0.008	^**^
C18:1 c6	0.36^b^	0.49^a^	0.42^a^	0.44^a^	0.43	0.007	^**^
C19:1 t7	NA	NA	NA	NA	NA
C20:1 c8	0.16^a^	0.06^c^	0.13^ab^	0.09^bc^	0.11	0.005	^**^
**FA group**							
∑ SFA	70.32	70.03	70.54	70.51	70.35	0.235	ns
∑ ECSFA	67.63	66.79	67.26	67.06	67.19	0.245	ns
∑ OCSFA	1.64^b^	2.00^a^	2.06^ab^	2.04^ab^	1.94	0.021	^**^
∑ BCSFA	1.04^b^	1.24^ab^	1.22^ab^	1.41^a^	1.23	0.032	^*^
∑ UFA	29.68	29.97	29.46	29.49	29.65	0.235	ns
∑ n-1 UFA	0.27^b^	0.31^a^	0.30^a^	0.32^a^	0.30	0.004	^*^
∑ n-3 UFA	0.50	0.67	0.74	0.74	0.66	0.024	ns
∑ n-5 UFA	0.66^c^	0.85^a^	0.76^b^	0.81^ab^	0.77	0.008	^**^
∑ n-6 UFA	3.78^ab^	4.41^a^	3.83^b^	4.28^ab^	4.08	0.066	^*^
∑ n-7 UFA	2.76^b^	3.79^a^	3.11^b^	3.58^a^	3.31	0.051	^**^
∑ n-9 UFA	21.04	19.10	19.96	18.99	19.77	0.201	ns
∑ n-12 UFA	0.67^b^	0.83^a^	0.76^a^	0.77^a^	0.76	0.011	^*^
∑ MUFA^14^	25.55	25.04	25.03	24.58	25.05	0.225	ns
∑ PUFA^15^	4.13	4.93	4.43	4.91	4.60	0.090	ns
∑ n-3 PUFA^16^	0.50	0.67	0.74	0.74	0.66	0.024	ns
∑ n-6 PUFA^17^	3.28^ab^	3.81^a^	3.31^b^	3.74^ab^	3.53	0.060	^*^
∑ CLA^18^	0.35	0.45	0.38	0.44	0.40	0.012	ns
∑ TFA^19^	1.31^c^	1.83^a^	1.43^bc^	1.63^ab^	1.55	0.030	^**^

NEC, Northeast China and Inner Mongolia; NC, North China; NWC, Northwest China; SC, South China; NA, not available.

1 Significances were declared at ^**^P < 0.01; ^*^P < 0.05; ns, *P* > 0.05. Different lowercase letters indicate a significant difference (*P* < 0.05).

2 SFA, saturated fatty acid, C4:0–C24:0 (including iso and anteiso).

3 ECSFA, even-chain saturated fatty acid, C4:0–C24:0.

4 OCSFA, odd-chain saturated fatty acid, C5:0–C23:0.

5 BCSFA, branched-chain saturated fatty acid, C13:0–C18:0 iso and anteiso.

6 UFA, unsaturated fatty acid, C10:1–C22:6.

7 n-1 UFA, omega-1 unsaturated fatty acid, the first double bond placed on the first carbon atoms counting from the methyl end.

8 n-3 UFA, omega-3 unsaturated fatty acid, the first double bond placed on the third carbon atoms counting from the methyl end.

9 n-5 UFA, omega-5 unsaturated fatty acid, the first double bond placed on the fifth carbon atoms counting from the methyl end.

10 n-6 UFA, omega-6 unsaturated fatty acid, the first double bond placed on the sixth carbon atoms counting from the methyl end.

11 n-7 UFA, omega-7 unsaturated fatty acid, the first double bond placed on the seventh carbon atoms counting from the methyl end.

12 n-9 UFA, omega-9 unsaturated fatty acid, the first double bond placed on the ninth carbon atoms counting from the methyl end.

13 n-12 UFA, omega-12 unsaturated fatty acid, the first double bond placed on the twelve carbon atoms counting from the methyl end.

14 MUFA, monounsaturated fatty acid, C10:1–C24:1.

15 PUFA, polyunsaturated fatty acid, C18:2–C22:6.

16 n-3 PUFA, omega-3 polyunsaturated fatty acid, contains more than one carbon-to-carbon double bond with the first double bond placed on the third carbon atoms counting from the methyl end.

17 n-6 PUFA, omega-6 polyunsaturated fatty acid, contains more than one carbon-to-carbon double bond with the first double bond placed on the sixth carbon atoms counting from the methyl end.

18 CLA, conjugated linoleic acid, C18:2 c9t11, C18:2 c9c11, C18:2 t9t11, C18:2 t10c12.

19 TFA, trans fatty acid, contains one or more than one trans carbon-to-carbon double bond and excludes conjugated linoleic acid.

### 3.3. Milk fatty acid nutritional quality indexes

Some FA indexes have been used to evaluate the nutritional quality of milk and are listed in Table 4.

**Table 4:** Nutritional indices for assessing fatty acids quality of retail milk samples.

**Health-related FA indices**	**NEC (*n* = 8)**	**NC (*n* = 51)**	**NWC (*n* = 13)**	**SC (*n* = 114)**	**Mean**	**SEM**	***P*-value^1^**
n-6/n-3 PUFA^2^	6.56^a^	6.63^ab^	5.54^b^	5.51^b^	6.06	0.13	^**^
P/S^3^	0.06	0.07	0.06	0.07	0.07	0.00	ns
AI^4^	2.83	2.66	2.84	2.75	2.77	0.04	ns
TI^5^	3.52	3.30	3.42	3.34	3.39	0.04	ns
HPI^6^	0.36	0.38	0.36	0.38	0.37	0.00	ns
h/H^7^	0.52	0.50	0.49	0.50	0.50	0.01	ns

NEC, Northeast China and Inner Mongolia; NC, North China; NWC, Northwest China; SC, South China.

1 Significances were declared at ^**^P < 0.01; ^*^P < 0.05; ns, *P* > 0.05. Different lowercase letters indicate a significant difference (*P* < 0.05).

2 n-6/n-3 PUFA = ∑ n-6 PUFA/∑ n-3 PUFA.

3 P/S, ∑ PUFA/∑ SFA.

4 AI, atherogenic index = (C12:0 + 4 × C14:0 + C16:0)/(∑ MUFA +∑ PUFA).

5 TI, thrombogenic index = (C14:0 + C16:0 + C18:0)/[(0.5 × ∑ MUFA + 0.5 × ∑ n-6 PUFA + 3 × ∑ n-3 PUFA) + ∑ n-3 PUFA/∑ n-6 PUFA].

6 HPI, health-promoting index = (∑ MUFA +∑ PUFA)/(C12:0 + 4 × C14:0 + C16:0).

7 h/H, hypo-/hypercholesterolemic ratio = (C18:1 n-9 + C18:2 n-6 + C20:4 n-6 + C18:3 n-3 + C20:5 n-3 + C22:5 n-3 + C22:6 n-3)/(C14:0 + C16:0).

n-6 and n-3 PUFAs induce proinflammatory and anti-inflammatory responses, respectively, and their ratios are related to a balanced diet (39–41). A balanced n-6/n-3 PUFA ratio allows for progressive brain, eyes, and heart development while reducing the risk of coronary heart disease and neurodegenerative disorders (42, 43). In the study, the n-6/n-3 PUFA content of milk in Northwest and South China was lower than that of milk from other regions. The decreased ratio indicated a desirable alteration between regional milk and benefited adults. To enable proper neuronal development and prevent most chronic disorders, a 1 or 2 n-6/n-3 PUFA ratio should be maintained (44, 45). In this study, the n-6/n-3 PUFA ratio of milk in different regions is 5.51–6.63, which is still a certain distance from the recommended balanced n-6/n-3 PUFA ratio, thus, further regulation of this composition is needed.

The P/S ratio is commonly used to assess the effect of dietary FA on CVH. The index is based on the research that dietary PUFAs are associated with decreased serum cholesterol and low-density lipoprotein content, while dietary SFAs are associated with increased serum cholesterol content (46). Therefore, the higher the P/S ratio, the more significant the inhibitory effect on the increase of blood cholesterol, and the more beneficial to cardiovascular health. In this study, there was no significant difference in the P/S ratio of milk from different regions, indicating that milk intake from different regions had no significant effect on CVH. The P/S ratio of milk ranged from 0.02 to 0.07 previously reported in the literature (22, 47). Our results were within the expected range of P/S index values, ranging from 0.06 to 0.07.

AI index characterized the atherogenic potential of dietary FA. The index shows the correlation between the pro-atherogenic FA (C12:0, C14:0, and C16:0) and the anti-atherogenic FA (UFA) (48). Thus, the lower the values of AI, the greater the proportion of anti-atherogenic fatty acids present in milk, and the more conducive to the maintenance of CVH. In this study, there was no significant difference in the values of AI of milk from different regions, indicating that intake of milk from different regions had no significant effect on the anti-atherosclerosis effect. The values of the AI index ranged from 1.37 to 5.13 reported in the previous literature (47, 49). Our results were within the expected range of AI index values, ranging from 2.66 to 2.84.

TI index was used to evaluate the tendency of dietary FA to thrombosis in blood vessels. This index is defined as the relationship between the pro-thrombotic FA (C12:0, C14:0, and C16:0) and the anti-thrombotic FA (MUFA, n-3 PUFA, and n-6 PUFA) (48). Specifically, the lower the values of the TI index, the greater the anti-thrombotic effect, and the better for CVH. In this study, there was no significant difference in the TI of milk from different regions, indicating that intake of milk from different regions had no significant effect on the anti-thrombosis effect. The values of the TI index ranged from 2.23 to 4.03 reported in the previous literature (50). Our results were within the expected range of AI index values, ranging from 3.30 to 3.52.

h/H ratio was commonly used to assess the cholesterolemic effect of dietary FA. The index is based on the research that dietary C18:1 and PUFA are associated with decreased serum cholesterol content, while dietary C12:0, C14:0, and C16:0 are associated with increased serum cholesterol content (51). Therefore, nutritionally speaking, the higher the h/H ratio, the more obvious the effect of inhibiting the increase of serum cholesterol content, and the more beneficial to CVH. In this study, there was no significant difference in the h/H ratio among the different regional milk samples, indicating that milk intake from different regions had no significant effect on human cholesterol metabolism. The h/H ratio range of milk reported in the literature is 0.32–0.74 (50). Our milk results were within the expected range of h/H ratio, ranging from 0.49 to 0.52.

HPI is the reciprocal of AI, which is mainly used to evaluate the impact of the fatty acid composition of dairy products on cardiovascular health (52). Therefore, the higher the HPI index, the more beneficial to CVH. In this study, there was no significant difference in the HPI among the different regional milk samples, indicating that the nutritional implications for CVH were similar among retail milk from different regions. Previous literature reported that the values of the HPI index ranged from 0.16 to 0.68 (20, 52). Our results were within the expected range of HPI index values, ranging from 0.36 to 0.38.

### 3.4. Potential implications on fatty acid intake via retail milk in China consumers

To determine whether the differences in milk FA composition from different geographic regions caused nutritionally meaningful differences, FA intake from retail milk was assessed in different regions (Table 5). Overall, FA intake was within similar ranges regardless of the region. The World Health Organization (WHO) has recommended SFA intake of <10% of energy intake, and the Chinese Nutrition Society has set an acceptable macronutrient distribution range (AMDR) for SFA intake as <8% of energy intake in children 4–17 years and <10% in adults over 18 years of age (21, 53). SFA from retail milk would provide either 33% of total SFA intake (based on recommended values by the WHO) or 33%−41% of total SFA intake (based on recommended values by the Chinese Nutrition Society). Stergiadis (22) reported that dairy fat contributed to approximately one-third of the maximum recommended intake of SFA in adult consumer diets across the UK dairy production systems, which is consistent with our findings. Although the Chinese Nutrition Society has not set a maximum intake of TFA, the WHO has recommended a TFA intake of <1% of energy intake (21, 53). Estimated TFA intake from retail milk throughout China is 6%−9% of the maximum recommended TFA daily intake, which would be well below the maximum values recommended by the WHO.

**Table 5:** Recommended dietary intakes of fatty acid with comparison to estimated intakes from retail milk.

**FA**	**Recommended dietary intakes**^**a**^ **(mg/d)**		**Estimated intakes from retail milk**^**b**^ **(mg/d)**				
	**Chinese Nutrition Society**	**World Health Organization**	**NEC (*****n*** = **8)**	**NC (*****n*** = **51)**	**NWC (*****n*** = **13)**	**SC (*****n*** = **114)**	**Mean**
SFA^c^	<18,162–22,703	<22,703	7,482 (7,085–7,876)	7,451 (6,884–8,026)	7,505 (7,186–8,024)	7,502 (6,885–8,374)	7,485 (6,884–8,374)
UFA^d^		36,324	3,158 (2,760–3,552)	3,189 (2,611–3,752)	3,134 (2,613–3,450)	3,137 (2,263–3,751)	3,155 (2,263–3,752)
n-6 PUFA^e^	5,676–20,432		349 (313–373)	405 (275–561)	352 (261–581)	398 (236–583)	376 (236–583)
n-3 PUFA^f^	1,135–4,541		53 (47–67)	72 (28–156)	78 (41–199)	79 (35–165)	71 (28–199)
LA^g^	9,081		267 (239–292)	272 (205–348)	225 (188–277)	266 (172–350)	258 (172–350)
ALA^h^	1,362		30 (27–36)	34 (17–56)	37 (28–62)	38 (22–62)	35 (17–62)
EPA+DHA^i^	250–2,000		7 (6–9)	14 (5–40)	14 (6–38)	14 (5–34)	12 (5–40)
TFA^j^		2,270	140 (113–186)	195 (134–290)	152 (80–254)	174 (89–254)	165 (80–290)

NEC, Northeast China and Inner Mongolia; NC, North China; NWC, Northwest China; SC, South China.

a Values displayed were based on an 8,400 kJ/d diet. FA intake (mg/d) was calculated from % kJ/d by converting total kJ to g (based on 1 g fat = 37 kJ) × 1,000. Recommended dietary intakes of FA (mg/d) = 8,400 (energy intake from diet kJ/d) × recommended dietary intakes of FA (% energy) ÷ 100 ÷ 37 (energy of per fat kJ/g fat) × 1,000.

b Estimated intakes from retail milk (mg/d) = 300 (milk intake g/d) × 1,000 × 3.8 (% contribution of fat from whole milk) ÷ 100 × 93.33 (correction factor representing % of FA in total milk fat) ÷ 100 × milk FA concentration (% of total FA) ÷ 100.

c SFA, saturated fatty acid, C4:0–C24:0 (including iso and anteiso).

d UFA, unsaturated fatty acid, C10:1–C22:6.

e n-6 PUFA, omega-6 polyunsaturated fatty acid, contains more than one carbon-to-carbon double bond with the first double bond placed on the sixth carbon atoms counting from the methyl end.

f n-3 PUFA, omega-3 polyunsaturated fatty acid, contains more than one carbon-to-carbon double bond with the first double bond placed on the third carbon atom counting from the methyl end.

g LA, linoleic acid, C18:2 c9c12.

h ALA, α-linolenic acid, C18:3 c9c12c15.

i EPA, eicosapentaenoic acid, C20:5 c5c8c11c14c17; DHA, docosahexaenoic acid, C22:6 c4c7c10c13c16c19.

j TFA, trans fatty acid, contains one or more than one trans carbon-to-carbon double bond and excludes conjugated linoleic acid.

Based on recommended UFA intake by the WHO, UFAs from retail milk in China would provide ~7% of the total UFA intake for consumers (21, 53). Furthermore, the Chinese Nutrition Society has suggested an AMDR of n-6 and n-3 PUFAs, expressed as % of energy intake, from 2.5 to 9% and from 0.5 to 2%, respectively. According to the results of the present study and current milk intake in China, retail milk throughout China would provide an intake of n-6 PUFA and n-3 PUFA from 2 to 7% and 2 to 6 % of AMDR, respectively. Additionally, the Chinese Nutrition Society has advised that the adequate intake (AI) of LA and ALA is 4 and 0.6% of energy intake, respectively. The consumption of retail milk would contribute ~3% of AI. The Chinese Nutrition Society has set the AMDR for EPA + docosahexaenoic acid (DHA) between 250 mg/d and 2,000 mg/d. Our study showed that the consumption of retail milk would provide an intake of EPA + DHA from 0.6 to 4.9% of AMDR. Due to the low content of EPA and DHA in retail milk and the low conversion efficiency of dietary ALA in humans (54), the supply of EPA and DHA from milk is extremely low; therefore, an additional supply of EPA and DHA from other foods is essential. Nutritional recommendations refer to the total diet rather than individual foods (21, 26). Although the current study estimated potential changes in FA intake from dairy products, any potential effects on human health are influenced by FA intake from other foods. Future research should investigate the nutritional roles of the individual components of milk.

## 4. Conclusion

We improved the GC-MS method for the simultaneous analysis of 82 FAs in milk samples with competitive sensitivity, suitable linearity, acceptable accuracy, and good precision. Analyses of regional effects indicated that the overall composition of milk FAs among different regions across China was numerically similar, and minor FAs showed little difference. When considering the retail milk FA composition and dairy fat intake in China, regional variations have a limited impact on FA consumption. Moreover, estimated intake of SFA and TFA via milk consumption accounted for approximately one-third and <10% of the maximum recommended values, respectively. The FA indices showed that the potential health effects of milk in different regions were inconsistent, which was indicated by the results of other FA indices, in addition to n-6/n-3 PUFA, which showed that regional differences in commercially available milk did not have much impact on cardiovascular health. After a reasonable assessment using indices with different emphases on health benefits, further systematic clinical trials should be conducted to determine the nutritional effects of milk.

## Data availability statement

The original contributions presented in the study are included in the article/Supplementary material, further inquiries can be directed to the corresponding authors.

## Author contributions

MC: conceptualization, methodology, formal analysis, investigation, writing—original draft, writing—reviewing and editing, and visualization. FW: conceptualization and writing—reviewing and editing. XW: formal analysis and writing—reviewing and editing. BS: methodology. JP: validation and writing—reviewing and editing. NZ: investigation and resources. YZ: supervision and resources. JW: writing—reviewing and editing, supervision, and conceptualization. All authors contributed to the article and approved the submitted version.
