# Supplementary material for: Updating the fatty acid profiles of retail bovine milk in China based on an improved GC-MS method: implications for nutrition
Source: Front Nutr. 2023 May 25;10:1204005. doi: 10.3389/fnut.2023.1204005 (PMC10248175; doi:10.3389/fnut.2023.1204005)
Supplement: Supplementary file 1 [file Data_Sheet_1.docx]

Supplementary Material

# Supplementary Tables

Table S1 The sample size and brand size of collected cow retail milk samples

Table S2 Linearity, sensitivity, and precision of the proposed method

Table S3 Recovery experiments of the proposed method

Table S1 The sample size and brand size of collected cow retail milk samples

| Province | Sample size | Brand size | Fat content % |
| --- | --- | --- | --- |
| *Northeast China and Inner Mongolia* | | | 3.2~4.2 |
| Inner Mongolia | 7 | 3 |  |
| Heilongjiang | 1 | 1 |  |
| *North China* | | | 3.3~3.6 |
| Beijing | 7 | 1 |  |
| Hebei | 8 | 1 |  |
| Shandong | 25 | 3 |  |
| Shanxi | 6 | 1 |  |
| Tianjin | 5 | 3 |  |
| *Northwest China* | | | 3.4~3.8 |
| Gansu | 9 | 2 |  |
| Shaanxi | 1 | 1 |  |
| Xinjiang | 3 | 3 |  |
| *South China* | | | 3.7~4.4 |
| Anhui | 4 | 1 |  |
| Fujian | 14 | 3 |  |
| Guangdong | 29 | 5 |  |
| Guizhou | 6 | 1 |  |
| Hubei | 8 | 1 |  |
| Hunan | 6 | 1 |  |
| Jiangsu | 11 | 3 |  |
| Shanghai | 4 | 1 |  |
| Sichuan | 11 | 3 |  |
| Yunnan | 7 | 2 |  |
| Zhejiang | 12 | 3 |  |
| Chongqing | 2 | 1 |  |
| *Total* | 186 | 44 | 3.8 |

Table S2 Linearity, sensitivity, and precision of the proposed method

| FAME^a^ | calibration curve regression equation (μg/L) | R^2^ | Linear range (μg/L) | LOQ^b^ (μg/L) | Inter-day RSD^c^ (%) | Intra-day RSD (%) |
| --- | --- | --- | --- | --- | --- | --- |
| C4:0 | y=0.10x+0.02 | 0.9999 | 400-100000 | 193.2 | 0.8 | 1.0 |
| C5:0 | y=1.40x+0.06 | 1.0000 | 100-10000 | 21.7 | 2.6 | 2.8 |
| C6:0 | y=1.88x+0.22 | 0.9999 | 100-100000 | 26.3 | 0.5 | 0.7 |
| C7:0 | y=4.04x+0.08 | 1.0000 | 100-10000 | 23.8 | 2.1 | 3.0 |
| C8:0 | y=2.61x+0.10 | 0.9999 | 100-100000 | 9.5 | 0.7 | 1.1 |
| C9:0 | y=1.94x+0.01 | 1.0000 | 100-10000 | 14.5 | 1.8 | 1.8 |
| C10:0 | y=2.81x+0.12 | 0.9995 | 50-100000 | 1.9 | 0.7 | 0.8 |
| C10:1 c4 | y=0.49x | 0.9998 | 541-21647 | 74.4 | NA | NA |
| C10:1 c3 | y=0.24x | 0.9996 | 541-21647 | 157.0 | NA | NA |
| C10:1 c9 | y=0.62x | 0.9996 | 541-21647 | 53.1 | 1.2 | 1.9 |
| C11:0 | y=2.64x+0.02 | 0.9993 | 100-100000 | 33.3 | 1.4 | 1.4 |
| C12:0 | y=2.70x+0.13 | 0.9996 | 50-100000 | 5.0 | 1.0 | 1.7 |
| C12:1 c5 | y=0.15x | 0.9999 | 535-21386 | 75.1 | NA | NA |
| C13:0 iso | y=0.56x | 1.0000 | 101-101000 | 29.5 | 1.4 | 4.6 |
| C12:1 c11 | y=0.15x | 0.9997 | 535-21386 | 175.1 | NA | NA |
| C13:0 anteiso | y=0.30x | 0.9999 | 116-23200 | 33.1 | NA | NA |
| C13:0 | y=0.44x-0.01 | 0.9991 | 100-100000 | 40.0 | 1.9 | 1.7 |
| C14:0 iso | y=1.78x | 0.9997 | 30-6000 | 25.5 | 1.8 | 2.4 |
| C14:0 | y=2.46x+0.07 | 0.9991 | 100-200000 | 14.5 | 1.0 | 1.7 |
| C15:0 iso | y=0.19x-0.01 | 1.0000 | 302-151000 | 35.6 | 2.0 | 2.0 |
| C14:1 t9 | y=0.19x-0.01 | 0.9993 | 500-50000 | 270.0 | 2.7 | 3.3 |
| C15:0 anteiso | y=0.10x | 0.9999 | 1110-555000 | 504.6 | 1.3 | 1.8 |
| C14:1 c9 | y=0.19x-0.01 | 0.9995 | 400-100000 | 111.1 | 1.1 | 1.7 |
| C15:0 | y=0.26x-0.01 | 0.9996 | 100-100000 | 29.4 | 1.3 | 1.9 |
| C16:0 iso | y=0.22x-0.01 | 0.9997 | 74-37000 | 37.0 | 1.0 | 1.5 |
| C15:1 t10 | y=0.20x-0.01 | 0.9997 | 500-50000 | 463.8 | NA | NA |
| C15:1 c10 | y=0.20x-0.01 | 0.9998 | 400-100000 | 222.2 | NA | NA |
| C16:0 | y=0.30x-0.01 | 0.9996 | 150-300000 | 18.8 | 1.1 | 2.0 |
| C16:1 t9 | y=0.18x-0.01 | 0.9998 | 500-50000 | 409.0 | 2.2 | 2.7 |
| C17:0 iso | y=0.25x-0.01 | 1.0000 | 138-138000 | 51.1 | 3.1 | 3.8 |
| C16:1 c7 | y=0.13x-0.01 | 0.9997 | 528-21104 | 175.2 | 4.1 | 4.5 |
| C16:1 c9 | y=0.18x-0.01 | 0.9999 | 400-200000 | 310.1 | 2.5 | 4.8 |
| C17:0 anteiso | y=0.25x | 0.9997 | 94-47000 | 39.2 | 3.1 | 2.8 |
| C17:0 | y=0.32x-0.02 | 0.9999 | 150-150000 | 42.9 | 0.8 | 1.3 |
| C17:1 t10 | y=0.24x-0.02 | 0.9998 | 1000-100000 | 771.0 | NA | NA |
| C18:0 iso | y=0.23x | 0.9994 | 80-8000 | 72.7 | 5.0 | 4.2 |
| C17:1 c10 | y=0.23x | 0.9998 | 1000-100000 | 694.0 | NA | NA |
| C18:0 | y=0.35x-0.03 | 0.9998 | 100-200000 | 33.3 | 0.9 | 1.1 |
| C18:1 t6 | y=0.26x-0.04 | 0.9998 | 1050-52482 | 406.8 | 1.6 | 3.8 |
| C18:1 t9 | y=0.27x-0.04 | 0.9997 | 1500-150000 | 929.5 | 4.2 | 4.2 |
| C18:1 t11 | y=0.26x-0.02 | 0.9997 | 1050-52482 | 779.3 | 2.7 | 4.5 |
| C18:1 c6 | y=0.26x-0.05 | 0.9990 | 1050-52482 | 337.4 | 4.9 | 4.9 |
| C18:1 c9 | y=0.26x-0.03 | 1.0000 | 1000-200000 | 952.4 | 3.4 | 3.5 |
| C18:1 c11 | y=0.25x-0.03 | 0.9999 | 1050-52482 | 268.3 | 3.6 | 2.6 |
| C18:1 c12 | y=0.15x-0.01 | 0.9999 | 500-50000 | 490.2 | 4.2 | 4.8 |
| C19:0 | y=0.64x-0.05 | 0.9995 | 100-10000 | 238.0 | 0.6 | 1.1 |
| C18:2 t9t12 | y=0.15x-0.03 | 1.0000 | 1000-100000 | 549.5 | 3.1 | 4.2 |
| C18:2 c9t12 | y=0.10x | 1.0000 | 500-50000 | 357.0 | 3.0 | 2.5 |
| C18:2 t9c12 | y=0.12x | 0.9997 | 500-20000 | 485.4 | 3.3 | 3.8 |
| C19:1 t7 | y=0.26x-0.03 | 0.9999 | 1000-50000 | 943.4 | NA | NA |
| C19:1 t10 | y=0.28x-0.04 | 1.0000 | 1000-100000 | 943.4 | NA | NA |
| C18:2 c9c12 | y=0.12x-0.03 | 0.9999 | 600-150000 | 493.8 | 0.8 | 1.1 |
| C19:1 c10 | y=0.17x-0.01 | 0.9994 | 500-10000 | 722.0 | NA | NA |
| C20:0 | y=4.12x-0.68 | 1.0000 | 1000-100000 | 492.6 | 1.2 | 1.6 |
| C18:3 c6c9c12 | y=1.90x-0.47 | 0.9999 | 1500-150000 | 634.8 | 2.1 | 3.5 |
| C20:1 t11 | y=0.23x-0.04 | 0.9999 | 1000-50000 | 794.3 | NA | NA |
| C18:3 c9c12c15 | y=2.63x-0.55 | 0.9998 | 1500-150000 | 681.2 | 1.2 | 1.9 |
| C20:1 c8 | y=0.12x-0.01 | 0.9990 | 1000-10000 | 990.1 | 3.3 | 4.0 |
| C20:1 c11 | y=0.28x-0.06 | 1.0000 | 1000-100000 | 952.3 | 2.4 | 4.0 |
| C18:2 c9t11 | y=0.38x-0.04 | 1.0000 | 520-52500 | 371.4 | 2.9 | 4.5 |
| C18:2 t10c12 | y=0.31x-0.02 | 1.0000 | 260-26250 | 255.7 | NA | NA |
| C18:2 c9c11 | y=0.30x-0.01 | 1.0000 | 1050-10500 | 552.6 | 3.1 | 4.2 |
| C21:0 | y=0.87x-0.11 | 1.0000 | 400-100000 | 381.0 | 2.4 | 4.0 |
| C18:4 c6c9c12c15 | y=0.45x-0.02 | 0.9993 | 500-10000 | 347.2 | NA | NA |
| C18:2 t9t11 | y=0.25x-0.01 | 0.9993 | 525-10500 | 410.2 | 2.7 | 4.2 |
| C20:2 c11c14 | y=1.88x-0.43 | 0.9998 | 1500-150000 | 783.7 | 2.6 | 4.1 |
| C22:0 | y=0.80x-0.08 | 0.9999 | 500-50000 | 485.4 | 2.1 | 3.1 |
| C20:3 c8c11c14 | y=1.76x-0.33 | 0.9999 | 1000-100000 | 693.5 | 1.6 | 2.6 |
| C22:1 t13 | y=0.19x-0.04 | 0.9999 | 1000-50000 | 840.3 | NA | NA |
| C20:3 c11c14c17 | y=2.49x-0.45 | 0.9999 | 1000-100000 | 537.1 | NA | NA |
| C22:1 c13 | y=0.21x-0.05 | 1.0000 | 1000-100000 | 980.4 | 1.8 | 2.3 |
| C20:4 c5c8c11c14 | y=1.98x-0.51 | 0.9999 | 600-150000 | 495.9 | 1.3 | 1.5 |
| C23:0 | y=2.91x-0.56 | 0.9998 | 400-100000 | 344.8 | 2.4 | 4.0 |
| C22:2 c13c16 | y=1.44x-0.42 | 0.9998 | 1000-100000 | 980.4 | NA | NA |
| C20:5 c5c8c11c14c17 | y=2.12x-0.51 | 0.9997 | 1000-100000 | 564.7 | 2.8 | 4.8 |
| C24:0 | y=2.54x-0.34 | 0.9995 | 500-50000 | 329.2 | 2.2 | 3.4 |
| C22:3 c13c16c19 | y=1.92x-0.18 | 0.9999 | 500-10000 | 315.3 | NA | NA |
| C24:1 c15 | y=0.18x-0.04 | 0.9998 | 500-50000 | 453.3 | NA | NA |
| C22:4 c7c10c13c16 | y=1.80x-0.50 | 0.9997 | 1000-100000 | 979.4 | NA | NA |
| C22:5 c4c7c10c13c16 | y=1.70x-0.50 | 0.9997 | 1000-100000 | 526.0 | 2.8 | 4.2 |
| C22:5 c7c10c13c16c19 | y=1.94x-0.58 | 0.9997 | 1000-100000 | 580.1 | 2.4 | 3.7 |
| C22:6 c4c7c10c13c16c19 | y=1.87x-0.55 | 0.9997 | 1000-100000 | 546.5 | NA | NA |

^a^ FAME, fatty acid methyl esters; ^b^ LOQ, limit of quantitation; ^c^ RSD, relative of standard deviation; NA, not available.

Table S3 Recovery experiments of the proposed method

| Items | Level (μg/mL) | | | | | | | |
| --- | --- | --- | --- | --- | --- | --- | --- | --- |
|  | Low (n=3) | |  | Middle (n=3) | |  | High (n=3) | |
|  | Recovery (%) | RSD (%) |  | Recovery (%) | RSD (%) |  | Recovery (%) | RSD (%) |
| TAG |  |  |  |  |  |  |  |  |
| C11:0^a^ | 104.2 | 6.8 |  | 101.9 | 1.6 |  | 106.3 | 0.5 |
| FA |  |  |  |  |  |  |  |  |
| C4:0^b^ | 81.4 | 5.0 |  | 82.3 | 4.8 |  | 82.0 | 6.1 |
| C6:0^b^ | 86.1 | 3.7 |  | 84.5 | 3.2 |  | 88.9 | 5.8 |
| C8:0^b^ | 92.2 | 3.2 |  | 92.4 | 1.2 |  | 94.2 | 1.3 |
| C10:0^c^ | 98.7 | 1.8 |  | 102.1 | 5.6 |  | 103.7 | 2.0 |
| C11:0^b^ | 106.3 | 1.4 |  | 102.2 | 4.4 |  | 102.2 | 0.6 |
| C12:0^c^ | 106.1 | 1.5 |  | 103.2 | 4.0 |  | 104.8 | 1.1 |
| C13:0^b^ | 105.0 | 1.8 |  | 103.1 | 3.2 |  | 104.0 | 0.8 |
| C14:0^b^ | 94.5 | 9.4 |  | 103.2 | 3.7 |  | 104.9 | 1.0 |
| C14:1 c9^b^ | 103.8 | 2.3 |  | 104.1 | 1.0 |  | 104.3 | 0.7 |
| C15:0^b^ | 107.5 | 1.8 |  | 102.2 | 2.2 |  | 105.1 | 0.4 |
| C15:1 c10^b^ | 97.8 | 0.5 |  | 103.2 | 1.1 |  | 105.0 | 0.5 |
| C16:0^c^ | 108.3 | 4.4 |  | 102.2 | 4.8 |  | 105.1 | 3.2 |
| C16:1 c9^b^ | 103.7 | 3.7 |  | 101.3 | 0.7 |  | 104.6 | 0.2 |
| C17:0^c^ | 101.4 | 3.4 |  | 104.8 | 2.0 |  | 106.6 | 0.5 |
| C17:1 c10^b^ | 99.9 | 1.5 |  | 95.0 | 1.4 |  | 101.5 | 0.5 |
| C18:0^c^ | 100.1 | 9.0 |  | 101.3 | 5.5 |  | 103.3 | 1.7 |
| C18:1 t9^b^ | 99.1 | 3.8 |  | 106.0 | 1.6 |  | 103.7 | 1.1 |
| C18:1 c9^c^ | 104.9 | 3.7 |  | 104.0 | 1.9 |  | 104.1 | 1.5 |
| C18:2 t9t12^b^ | 105.4 | 1.5 |  | 105.5 | 1.0 |  | 104.2 | 0.8 |
| C18:2 c9c12^b^ | 103.4 | 5.1 |  | 104.0 | 1.2 |  | 101.8 | 0.5 |
| C20:0^c^ | 101.6 | 2.0 |  | 101.3 | 1.4 |  | 104.3 | 0.3 |
| C18:3 c6c9c12^c^ | 104.9 | 0.4 |  | 101.6 | 0.8 |  | 105.0 | 0.1 |
| C18:3 c9c12c15^b^ | 103.4 | 1.4 |  | 102.8 | 1.2 |  | 105.0 | 0.1 |
| C20:1 c11^b^ | 105.8 | 0.5 |  | 99.6 | 2.7 |  | 103.2 | 0.9 |
| C21:0^b^ | 104.6 | 1.6 |  | 101.4 | 0.9 |  | 103.1 | 1.6 |
| C20:2 c11c14^b^ | 104.4 | 0.4 |  | 103.2 | 1.7 |  | 104.5 | 1.1 |
| C22:0^c^ | 104.4 | 2.1 |  | 104.3 | 0.9 |  | 101.5 | 1.2 |
| C20:3 c8c11c14^b^ | 101.2 | 0.6 |  | 102.6 | 1.1 |  | 102.8 | 1.0 |
| C20:3 c11c14c17^b^ | 103.3 | 2.4 |  | 103.2 | 1.7 |  | 103.6 | 0.4 |
| C22:1 c13^b^ | 102.5 | 6.1 |  | 102.1 | 6.3 |  | 103.9 | 2.6 |
| C20:4 c5c8c11c14^b^ | 104.1 | 1.0 |  | 100.3 | 2.1 |  | 103.7 | 0.4 |
| C23:0^b^ | 103.8 | 0.7 |  | 102.0 | 1.0 |  | 102.9 | 0.8 |
| C22:2 c13c16^b^ | 102.6 | 1.0 |  | 100.2 | 1.5 |  | 103.6 | 1.6 |
| C20:5 c5c8c11c14c17^b^ | 103.8 | 1.6 |  | 102.4 | 0.7 |  | 103.0 | 2.7 |
| C24:0^c^ | 101.0 | 1.0 |  | 102.1 | 1.5 |  | 105.7 | 0.4 |
| C24:1 c15^b^ | 103.2 | 1.4 |  | 104.5 | 3.3 |  | 105.1 | 1.6 |
| C22:4 c7c10c13c16^b^ | 105.5 | 0.4 |  | 102.8 | 1.0 |  | 103.1 | 1.3 |
| C22:5 c4c7c10c13c16^b^ | 105.1 | 1.3 |  | 102.0 | 1.4 |  | 104.5 | 0.7 |
| C22:5 c7c10c13c16c19^b^ | 103.8 | 0.4 |  | 104.2 | 1.4 |  | 104.3 | 0.2 |
| C22:6 c4c7c10c13c16c19^b^ | 103.3 | 1.1 |  | 103.3 | 2.6 |  | 103.7 | 1.1 |

^a^ Low, middle, and high spiked concentrations were 25 μg/mL, 100 μg/mL, and 250 μg/mL, respectively.

^b^ Low, middle, and high spiked concentrations were 20 μg/mL, 80 μg/mL, and 200 μg/mL, respectively.

^c^ Low, middle, and high spiked concentrations were 40 μg/mL, 160 μg/mL, and 400 μg/mL, respectively.
